# Ammonium as a Driving Force of Plant Diversity and Ecosystem Functioning: Observations Based on 5 Years' Manipulation of N Dose and Form in a Mediterranean Ecosystem

**DOI:** 10.1371/journal.pone.0092517

**Published:** 2014-04-02

**Authors:** Teresa Dias, Adelaide Clemente, Maria Amélia Martins-Loução, Lucy Sheppard, Roland Bobbink, Cristina Cruz

**Affiliations:** 1 Centro de Biologia Ambiental, Faculdade de Ciências da Universidade de Lisboa, Lisboa, Portugal; 2 Museu Nacional de História Natural e da Ciência, Jardim Botânico, Lisboa, Portugal; 3 Centre for Ecology and Hydrology – Edinburgh, Bush Estate, United Kingdom; 4 B-Ware Research Centre, Radboud University Nijmegen, Nijmegen, The Netherlands; North Carolina State University, United States of America

## Abstract

Enhanced nitrogen (N) availability is one of the main drivers of biodiversity loss and degradation of ecosystem functions. However, in very nutrient-poor ecosystems, enhanced N input can, in the short-term, promote diversity. Mediterranean Basin ecosystems are nutrient-limited biodiversity hotspots, but no information is available on their medium- or long-term responses to enhanced N input. Since 2007, we have been manipulating the form and dose of available N in a Mediterranean Basin maquis in south-western Europe that has low ambient N deposition (<4 kg N ha^−1^ yr^−1^) and low soil N content (0.1%). N availability was modified by the addition of 40 kg N ha^−1^ yr^−1^ as a 1∶1 NH_4_Cl to (NH_4_)_2_SO_4_ mixture, and 40 and 80 kg N ha^−1^ yr^−1^ as NH_4_NO_3_. Over the following 5 years, the impacts on plant composition and diversity (richness and evenness) and some ecosystem characteristics (soil extractable N and organic matter, aboveground biomass and % of bare soil) were assessed. Plant species richness increased with enhanced N input and was more related to ammonium than to nitrate. Exposure to 40 kg NH_4_^+^-N ha^−1^ yr^−1^ (alone and with nitrate) enhanced plant richness, but did not increase aboveground biomass; soil extractable N even increased under 80 kg NH_4_NO_3_-N ha^−1^ yr^−1^ and the % of bare soil increased under 40 kg NH_4_^+^-N ha^−1^ yr^−1^. The treatment containing less ammonium, 40 kg NH_4_NO_3_-N ha^−1^ yr^−1^, did not enhance plant diversity but promoted aboveground biomass and reduced the % of bare soil. Data suggest that enhanced NH_y_ availability affects the structure of the maquis, which may promote soil erosion and N leakage, whereas enhanced NO_x_ availability leads to biomass accumulation which may increase the fire risk. These observations are relevant for land use management in biodiverse and fragmented ecosystems such as the maquis, especially in conservation areas.

## Introduction

High biodiversity can stabilize ecosystems through functional complementarities, which can buffer the impacts of environmental change [1], [2]. Biodiversity and ecosystem functions are influenced by several drivers (*e.g.* land use change, increased nutrient availability), but ecosystem responses (and mechanisms) to those drivers remain unclear [3], especially responses of ecosystems under persistent anthropogenic influence such as enhanced nitrogen (N) deposition.

Enhanced N availability has been acknowledged as a global and increasing threat to biodiversity [4]–[7] and ecosystem function [8], [9]. However, most of our knowledge of the impacts of increased N availability on ecosystems comes from northern Europe and America [6]. Mediterranean-type ecosystems appear on the ‘neglected ecosystems list’ [6], [10] despite being a global conservation priority [11], [12], rivalling tropical rainforests [12], [13]. In fact, not much is known about the impacts of increased N availability on Mediterranean-type ecosystems, other than those found in California [10], [14]. Ecosystems in the Mediterranean Basin that have experienced intensive human development and impact for millennia [15], and where N deposition is expected to increase threefold by 2050 [5], [16] are particularly deserving of study.

The most distinctive features of Mediterranean-type ecosystems likely to influence responses to increased N availability are: climate (highly seasonal, with warm dry summers that contrast with cool rainy winters); soils (low nutrient levels and organic matter and high contents of bases such as carbonates); dominance of dry N deposition; asynchrony between N availability and biological activity [10]; and spatial and temporal heterogeneity [17]. On the basis of these distinguishing features, it seems likely that Mediterranean-type ecosystems could respond rather differently from north temperate ones. Even within Mediterranean-type ecosystems, differences in soil fertility [13] and phosphorus (P) availability in particular can undermine extrapolations from Californian ecosystems to those in the Mediterranean Basin [10]. For these reasons, in 2007 an N-manipulation (dose and form) field experiment was established in a severely nutrient-limited Mediterranean Basin maquis. Contrasting with most studies (north temperate systems [6]–[9] and Mediterranean Basin [18], [19]), within one year plant richness increased and changes in plant community composition were observed [20]. Recently, Forest et al. [3] provided data supporting the hypothesis that long-term impacts of N enrichment on ecosystem functioning depend strongly on biodiversity changes, especially non-random changes in species composition. As a result, our observation raises key questions:

- Is this N-driven increase in plant richness transient? This is especially relevant since N deposition in most European ecosystems has already reached a threshold, beyond which diversity has declined [21]. Accordingly, given our N doses, we expected that after 5 years, plant diversity would have stabilized or be in decline.
- Are there consistent plant responders to increased N availability in Mediterranean maquis? We hypothesized that the changes in plant cover and species composition along the ecological succession [22] may confound the identification of consistent plant responders to enhanced N availability.
- Are the N-driven changes in diversity linked with plant productivity? Experiments that directly manipulate species diversity often report a positive impact on productivity, whereas observations of natural communities reveal more complex relationships [2], and nutrient additions generally increase productivity but decrease diversity [23]. In our case, because we expected that the N-benefited species would be small, short-lived plants [24], [25] that contribute little to ecosystem functioning, even if plant richness continued to increase, we did not expect an increase in productivity.

Inherent to all these questions is the need to understand whether the form of N matters more than the dose. This is especially relevant since the co-existing plant species occupy distinct N niches, with ammonium (NH_4_^+^) availability as a determinant [17], [26], so that the plant's response to enhanced N availability may vary according to the N form irrespective of the dose.

We report 5 years of results from an N-manipulation field experiment, focusing on N-driven changes among the plant community and soil characteristics in a Mediterranean Basin maquis. Specifically, we focused on the following ecosystem features: vascular plant community (composition, richness and evenness), aboveground biomass (standing biomass and litter production), soil extractable N (NH_4_^+^ and nitrate – NO_3_^−^) and organic matter, and percentage of bare soil.

## Materials and Methods

### Study site

**Figure 1:**
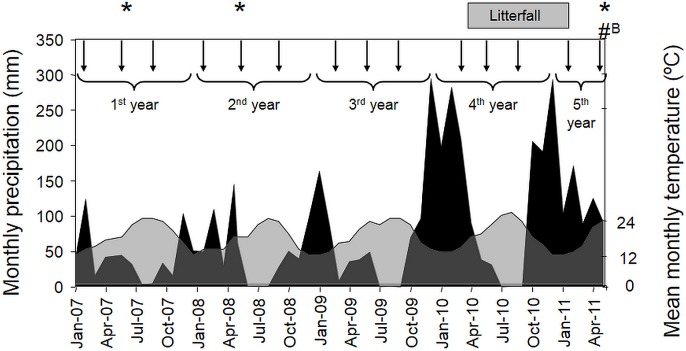
Weather conditions and main experimental events over the experimental period. Mean monthly temperature (light grey) and total monthly precipitation (black). Arrows represent the time of N additions from January 2007 to June 2011. Asterisks refers to the occasions of plant community assessments (2007, 2008 and 2011). Litterfall was collected from April to December 2010. “#B” refers to the time of aboveground biomass sampling (June 2011).

We are grateful to Arrábida Natural Park for making the experimental site available and allowing the N manipulation experiment to which this paper refers. The study site (38°29′N - 9°1′W) is located in Serra da Arrábida in the Arrábida Natural Park, Portugal (a Natura 2000 site - PTCON0010 Arrábida/Espichel), which is within the sub-humid thermomediterranean bioclimatic domain [27]. According to records (1971–2000 - Instituto Nacional de Meteorologia e Geofísica), mean annual precipitation is 730 mm; mean maximum temperature, 27.8°C (August); and mean minimum temperature, 8.1°C (January). Over the experimental period (2007–2011), mean annual precipitation was 870 mm (±231 mm, SE); mean maximum temperature, 21.6°C (±0.7°C, SE); and mean minimum temperature, 12.9°C (±0.5°C, SE). Total monthly precipitation and mean monthly temperatures over the experimental period are shown in Figure 1.

The site is located on a southeast-facing slope (5%) at 130 m altitude, which is protected from public access and has not been managed in recent decades. The soils of Serra da Arrábida are classified, according to the FAO system, as calcic rhodo-chromic luvisols and calcareous chromic cambisols [28]. The soil is skeletal (topsoil layer of approximately 15 cm) with a bulk density of 1.3 g cm^−3^. Silt predominates (50%), while sand and clay contents are 32% and 18% (silt-sand-loam texture).

Mediterranean maquis vegetation dominates the site, comprising closed vegetation: mainly shrubs with few annuals and some geophytes and normally with some trees, some of which may be in shrub form (Eunis class F5.2 – Mediterranean maquis). The standing community developed after a fire event in summer 2003, four years before the first N addition of this experiment. The dominant plant species was a Cistaceae, *Cistus ladanifer* L. [20], [29]. Other abundant plant species were *Erica scoparia* L. (Ericaceae), *Calluna vulgaris* (L.) Hull (Ericaceae), *Genista triacanthos* Brot. (Fabaceae), *Ulex densus* Welw. ex Webb (Fabaceae), *Dittrichia viscosa* L. (Asteraceae) and *Myrtus communis* L. (Myrtaceae). Herbaceous species, of which many were annual plants, comprised ≈10% of the total plant cover [20].

### Experimental design and fertilization schedule

During the experimental period (2007–2011), estimated background N deposition ranged between 2.9 kg ha^−1^ yr^−1^ (1.8 kg NO_x_+1.1 kg NH_y_) in 2008 and 3.8 kg ha^−1^ yr^−1^ (2.2 kg NO_x_+1.6 kg NH_y_) in 2010. These values were estimated based on the model used by the European Monitoring and Evaluation Programme (grid location: x = 53 and y = 4 - http://www.emep.int/mscw/index_mscw.html). The chosen N doses were high enough to simulate ‘worst case’ scenarios of N enrichment in this type of habitat, but lower than the N deposition reported for highly N polluted areas in Mediterranean-type ecosystems [30], [31]. The N forms mimicked the most likely N pollution scenarios within the Mediterranean Basin (*e.g.* agricultural sources alone or combined with urban/industrial sources). Control plots received no added N, while there were three N treatments: **40A** received 40 kg NH_4_^+^-N ha^−1^ yr^−1^ as a 1∶1 mixture of NH_4_Cl and (NH_4_)_2_SO_4_; **40AN** received 20 kg NH_4_^+^-N ha^−1^ yr^−1^ and 20 kg NO_3_^−^-N ha^−1^ yr^−1^ as NH_4_NO_3_; and **80AN** received 40 kg NH_4_^+^-N ha^−1^ yr^−1^ and 40 kg NO_3_^−^-N ha^−1^ yr^−1^ as NH_4_NO_3_. Thus the 40A and 40AN treatments provided the same N dose, while 40A and 80AN provided the same NH_4_^+^ dose. To prevent N ‘contamination’ through runoff from the N-plots, the experimental plots were randomly distributed in three rows across the slope, with the controls being located in the top row.

Beginning in January 2007, the dry N salts were homogenously added, by hand, sprinkled over the soil surface, in three equal applications over the year: mid-autumn/winter, spring and summer (Fig. 1). Each treatment was replicated three times (3 plots of 400 m^2^ each). To restrict boundary effects and dilution processes, all measurements, analyses and sample collection were performed within the central 100 m^2^ square.

### Plant diversity and % of bare soil

The composition of the vascular plant community was assessed in June 2007, May 2008 [20] and June 2011 within one 5×5 m square per experimental plot (within the internal 100 m^2^). Percentages of species cover calculated from the total projected crown area and of bare soil (as a measure of erosion potential) were recorded. Plant species observed in the three community assessments are grouped by life form in Table S1 using the Flora Digital de Portugal database (http://jb.utad.pt/pesquisa). This classification was considered more informative than others (*e.g.* by forb, grass, shrub and tree) because it provides some clues to plant size and life cycle. From the vascular plant community assessments in 2007 and 2011, it was possible to calculate plant richness and evenness [32] for the first and fifth springs of the experiment.

### Soil extractable N and organic matter

Soil inorganic N pools were measured to estimate soil N retention (the lower the soil inorganic N pools, the higher the N retention and vice versa), and organic matter was measured as a proxy for belowground C sequestration. Soil was sampled from the four corners and the centre of the central 100 m^2^ square of each plot. Soil samples (2 cm diameter and 15 cm depth) were removed, sieved (2 mm) and stored at 4°C until analysis. Sampling took place in May 2007 (first spring) and 2011 (fifth spring of the N additions). Individual soil samples (five per plot) were analysed for nitrate (NO_3_^−^-N [33]), ammonium (NH_4_^+^-N [34]), pH (H_2_O extract) and organic matter [20]. Soil inorganic N (N_in_) was the sum of the water-extracted NH_4_^+^ and NO_3_^−^. NO_3_^−^, NH_4_^+^ and N_in_ were expressed as μg N per gram of dry soil. Bulk soil samples (equal mixtures of the five soil samples from each experimental plot) were dried (at 60°C until constant mass), ground (MM 2000) and used for quantifying total soil N and C, and hence the C/N ratio, by dry combustion using an elemental analyser (EuroVector, Italy).

### Aboveground biomass

Aboveground biomass included standing biomass and leaf litter production. The standing plant biomass was determined in June 2011 (the fifth spring of N additions – shown in Fig. 1) by removing all aboveground plant biomass within three randomly located 1 m^2^ squares per plot. Plant biomass was dried to constant mass at 60°C. Litterfall was assessed using litter traps (1.5 mm mesh screen with 0.04 m^2^ collecting surface; at ca. 15 cm above the ground) placed under the canopy of five *C. ladanifer* shrubs (located close to the four corners and at the centre of the internal 100 m^2^ square) in each plot. Litter was collected fortnightly from April to December 2010 (shown in Fig. 1) and weighed after drying to constant mass at 60°C. Given that the vegetation is dominated by summer semi-deciduous species that shed most of their leaves and twigs in the summer [17], [35], the litterfall collected between April and December 2010 was considered to represent the annual fall (Fig. 4-b). Since *C. ladanifer*'s aboveground biomass did not respond to the N addition treatments (data not shown) and litterfall in Californian ecosystems was not significantly affected by five years of 50 kg NH_4_NO_3_-N ha^−1^ y^−1^ additions [36], no changes in litterfall were also assumed in our study. The amount of biomass that was produced and ‘lost’ every year through litterfall was estimated by multiplying this annual value by four, representing years 2007 to 2010 (2011 was not considered since most leaf shedding occurs in summer/autumn). The sum of this value and the standing aboveground plant biomass was considered to correspond to the total aboveground biomass produced by the plant community during the experiment.

### Derived variables and statistics

The cumulative N, NH_4_^+^ and NO_3_^−^ load (estimated N deposition +N additions) at each plant assessment were calculated using the above-mentioned EMEP estimates and assuming that N, NH_y_ and NO_x_ were deposited homogenously over the year (Fig. 3).

Summary statistics of soil properties, plant species and community responses (richness, evenness, cover, biomass and litterfall) of the various N additions were compared. Two-way ANOVA was applied to determine if there were significant interactions between time and treatment for soil and plant variables. Differences per treatment in biomass and litterfall were analysed by a one-way ANOVA. Both types of ANOVA were followed by a Bonferroni test (*p*<0.05 or *p*<0.1), or by a Games-Howell test whenever homogeneity of variances was not confirmed by the Levene's test. Between treatment differences for change in plant cover were analysed by a one-way ANOVA (followed by a Bonferroni test *p*<0.1) and a Kruskal-Wallis test (*p*<0.1) for normal and non-normal samples respectively (Table S1). Linear correlations between plant richness and cumulative N, NH_4_^+^ and NO_3_^−^ were also studied (Pearson's correlations). Correlation between plant richness and cumulative NH_4_^+^ and that between plant richness and cumulative NO_3_^−^ were compared using the Steiger's Z test (*p*<0.05). In all cases, analyses were performed to ensure that the assumptions regarding the tests' application were not violated. SPSS software, version 20.0, was used for all tests.

## Results

### Impacts of the N treatments on plant diversity

**Figure 2:**
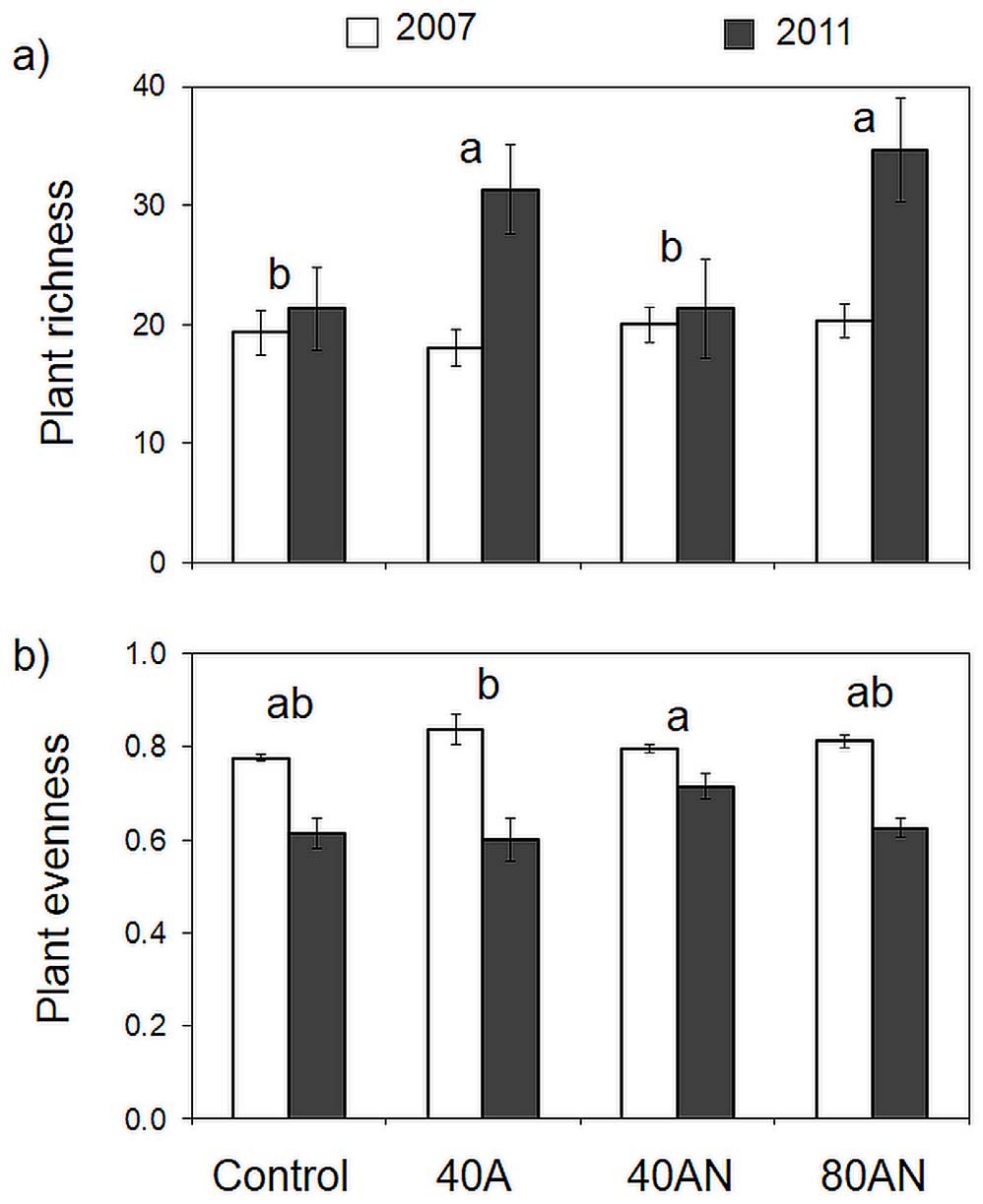
Impacts of the N treatments on vascular plant diversity. Response of the vascular plant community to the N treatments (Control, 40A, 40AN and 80AN), in terms of plant richness (a) and evenness (b). Community assessments were performed in the first and fifth springs of N additions: 2007 and 2011 respectively. Different letters refer to statistically significant differences between treatments (two-way ANOVA *p*<0.05 followed by a Bonferroni test; there were no significant interactions between treatment and time). Bars represent the mean (n = 3 experimental plots per treatment) ±SE.

**Figure 3:**
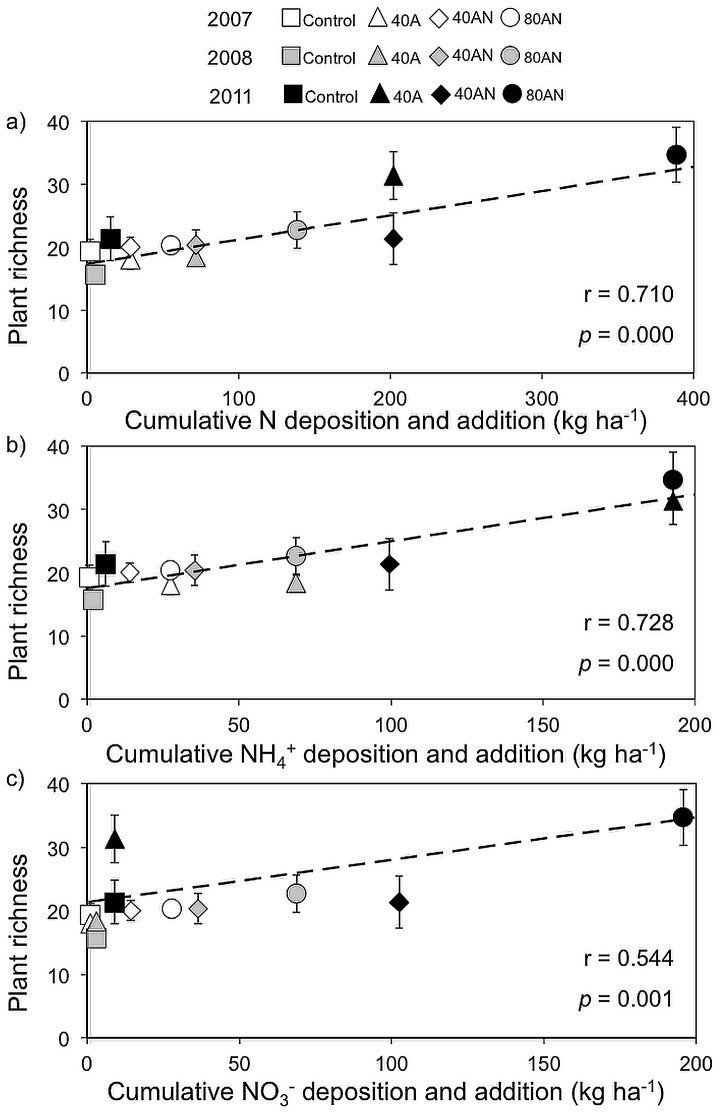
Relation between cumulative N and plant richness. Pearson's correlations between plant richness (number of vascular plant species per 25 m^2^) and cumulative N (a), NH_4_^+^ (b) and NO_3_^−^ (c) over the experiment, accounting for estimated background deposition (EMEP- see material and methods). Correlation between plant richness and cumulative NH_4_^+^ differed significantly from that between plant richness and cumulative NO_3_^−^ [Steiger's Z test (*p*<0.05)]. Symbols represent the mean (n = 3 experimental plots per treatment and per year) ±SE, but correlations were based on the individual values (n = 36).

As usual in the Mediterranean region, the annual rainfall was highly variable (ranging from 513 mm in 2007 and 1541 mm in 2010), with rainfall being greatest during the last two rainy seasons of the experiment (2009/2010 and 2010/2011) (Fig. 1). Initially (spring 2007), richness and evenness were similar in all treatments (Fig. 2). Between treatment differences were, however, evident after one year [20], and remained throughout (Table S1 and Fig. 2). Richness and composition changed the most (≈60% in relation to the control) in treatments receiving 40 kg NH_4_^+^-N ha^−1^ yr^−1^ (40A and 80AN – Fig. 2 and Table S1). In contrast, between 2007 and 2011, plant species richness (Fig. 2-a) did not change in the control or 40AN plots. Linear, positive and significant correlations were found between plant richness and cumulative N, NH_4_^+^ and NO_3_^−^ (Fig. 3). However, the correlation between plant richness and cumulative NH_4_^+^ addition was higher than that between plant richness and cumulative NO_3_^−^. During the same period, plant evenness decreased with time, decreasing least in the 40AN plots (Fig. 2-b).

**Figure 4:**
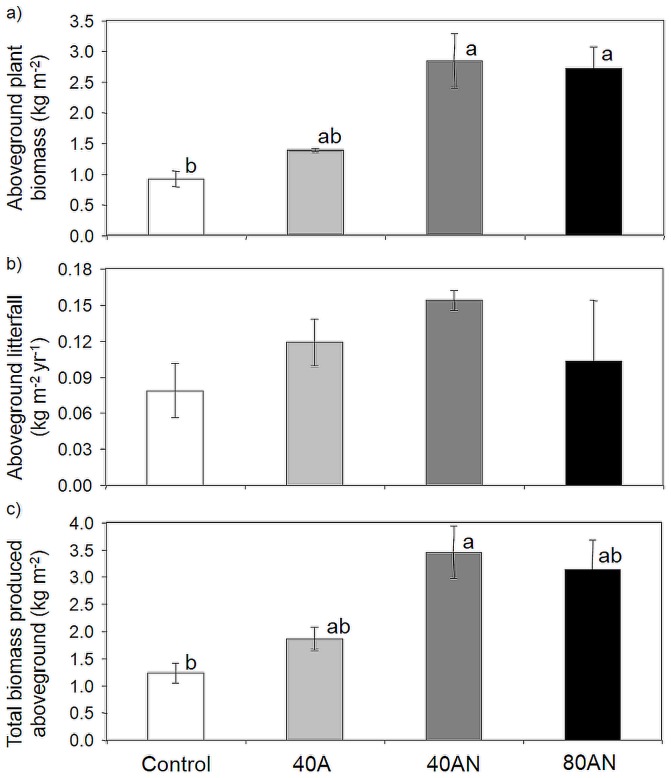
Impacts of the N treatments on plant biomass. Aboveground plant biomass (a), estimated litterfall production per year (b) and total aboveground biomass (sum of the standing biomass and the cumulative litterfall produced on the previous four years - c) according to the N treatments (Control, 40A, 40AN and 80AN). Aboveground plant biomass was harvested in June 2011 (the fifth spring of the experiment) from three 1-m^2^ squares per experimental plot. Litterfall was collected from April to December 2010 using litter traps (five 0.04 m^2^ per experimental plot). Different letters refer to statistically significant differences between treatments (ANOVA *p*<0.05 followed by a Bonferroni test). Bars represent the mean (n = 3 experimental plots per treatment) ±SE.

Plant community composition changed (between 2007 and 2011) as a result of both the ongoing post-fire ecological succession, indicated by the changes in the controls, and the N additions (Table S1). The life forms that were most responsive to N, and to NH_4_^+^ in particular, were small and ephemeral plants (geophytes, hemicryptophytes and therophytes, Table S1). Initially (2007–2008), *Dittrichia viscosa* benefited most from the increased N (irrespective of dose and form), while *Cistus ladanifer* was affected (negatively impacted) by 80AN. In the longer-term (2007–2011), *C. ladanifer* continued to respond negatively to 80AN, while *Ulex densus* was affected by 40A. These N-affected (negatively impacted by N) species were perennial shrubs. Small species with short life cycles benefited most from N (Table S1): *Hypericum* sp (hemicryptophytes), *Gastridium ventricosum* (annual) and *Sonchus* sp (annual).

Based on a consistent response over 5 years of N addition (Table S1), groups of species that responded similarly to the N dose or form were identified (Tables 1 and S2). No species showed a preference for low N availability, *i.e.* none significantly reduced its cover or disappeared from all the N treatments while remaining in the control plots. The covers of *D. viscosa* and *Sonchus* sp. decreased in all plots, although N appeared to offset some of the cover loss that was observed in the controls. The presence of *Carlina corymbosa*, *Gladiolus illyricus* ssp. *reuteri* and *Galium* sp. may indicate increased N availability, while that of *Salvia sclareoides*, *Asphodelus ramosus*, *Blackstonia perfoliata* and *Dactylis glomerata* may indicate high N availability (associated with the 80AN treatment). In terms of response to the form of N, *Pulicaria odora* appears to prefer NO_3_^−^ (only present in 40AN and 80AN plots), while *Sanguisorba hybrida* appears to avoid NO_3_^−^ (decreased cover in 40AN and 80AN), consistent with being characteristic of later phases of succession. Finally, *Rubia peregrina* and *Brachypodium phoenicoides* appear to prefer high NH_4_^+^ availability (associated with 40A and 80AN), whereas *Anemone palmate* appears to be rather sensitive to NH_4_^+^ (found in neither 40A nor 80AN).

**Table 1:** Plant species potentially indicative of the N dose and form.

		Benefited	Affected
**N dose**	**40 kg N ha^−1^ yr^−1^**	*- Carlina corymbosa*	
		*- Gladiolus illyricus ssp reuteri*	
		*- Galium sp*	
	**80 kg N ha^−1^ yr^−1^**	*- Salvia sclareoides*	*- Cistus ladanifer*
		*- Asphodelus ramosus*	
		*- Blackstonia perfoliata*	
		*- Dactylis glomerata*	
**N form**	**NO_3_^−^**	*- Pulicaria odora*	*- Sanguisorba hybrida*
	**NH_4_^+^**	*- Rubia peregrina*	*- Anemone palmata*
		*- Brachypodium phoenicoides*	

Plant species that responded consistently (over 5 years of N addition treatments) to the N dose and/or form (Tables S1 and S2).

### Impacts of N on ecosystem processes

N additions did not affect total N or C concentrations after one and five years (Tables 2 and S3). Soils contained very little N (0.1%) or C (∼2%), resulting in a C/N ratio <20. N treatments did increase soil available N and organic matter. In relation to the N dose, control plots contained significantly lower concentrations than the 80AN treatment plots, and plots receiving 40 kg N ha^−1^ yr^−1^ (40A and 40AN) showed intermediate levels. Soil NO_3_^−^ and soil inorganic N (these two variables were highly correlated, r = 0.98, *p*<0.001) increased over time. The soil also acidified over the course of the experiment, independent of treatment (Tables 2 and S3).

**Table 2:** Impact of the N treatments on soil properties.

Soil properties		Control	40A	40AN	80AN
N (%)	2007	0.1±0.0	0.1±0.0	0.1±0.0	0.1±0.0
	2011	0.1±0.0	0.1±0.0	0.1±0.0	0.1±0.0
C (%)	2007	1.6±0.2	1.6±0.3	1.8±0.1	2.0±0.6
	2011	1.8±0.1	1.9±0.2	2.1±0.1	2.2±0.3
C/N ratio	2007	18.5±0.8	16.6±0.1	17.4±1.9	16.6±1.0
	2011	18.6±0.4	17.4±0.7	18.1±0.5	17.6±0.8
	*	b	ab	ab	a
N_in_	2007	5.9±0.9	9.4±2.0	9.6±2.1	10.5±1.9
(μg g^−1^)	2011	7.9±1.3	12.2±1.9	12.9±1.6	19.2±2.5
	*	b	ab	ab	a
NO_3_^−^-N	2007	5.6±0.8	8.3±1.9	8.9±2.2	8.1±1.7
(μg g^−1^)	2011	7.3±1.3	9.8±1.1	12.0±1.5	17.4±2.3
	*	b	ab	ab	a
NH_4_^+^-N	2007	0.3±0.1	1.1±0.3	0.7±0.1	2.4±0.3
(μg g^−1^)	2011	0.6±0.1	2.3±1.0	0.9±0.1	1.8±0.5
	*	b	ab	ab	a
OM (%)	2007	5.7±0.7	5.8±0.3	6.4±0.3	7.0±1.2
	2011	4.9±0.5	6.5±0.3	7.0±0.5	7.8±0.5
	*				
pH (H_2_O)	2007	5.8±0.2	6.5±0.3	6.4±0.1	6.6±0.4
	2011	5.1±0.2	4.9±0.3	5.1±0.1	5.6±0.3

Soil surface (0–15 cm) properties [total N and C, C/N ratio, concentrations of N_in_ – extractable inorganic N, NO_3_^−^ – nitrate, and NH_4_^+^ – ammonium, OM – organic matter, and pH (H_2_O)], in the first (May 2007) and fifth (May 2011) springs of the experiment, according to the N addition treatment (Control, 40A, 40AN and 80AN). Different letters refer to significant differences between treatments (ANOVA *p*<0.05 followed by a Bonferroni test), while * refers to significant differences between 2007 and 2011 (there were no significant interactions between time and treatment, *p*<0.05 – Table S3). Values represent the mean (n = 3 experimental plots per treatment) ±SE.

The addition of 40AN and 80AN increased aboveground standing biomass over the control (Fig. 4-a). When estimated leaf litter (Fig. 4-b) produced between 2007 and 2011 is taken into account, aboveground biomass produced by the 40AN plants also exceeded controls (Fig. 4-c).

**Figure 5:**
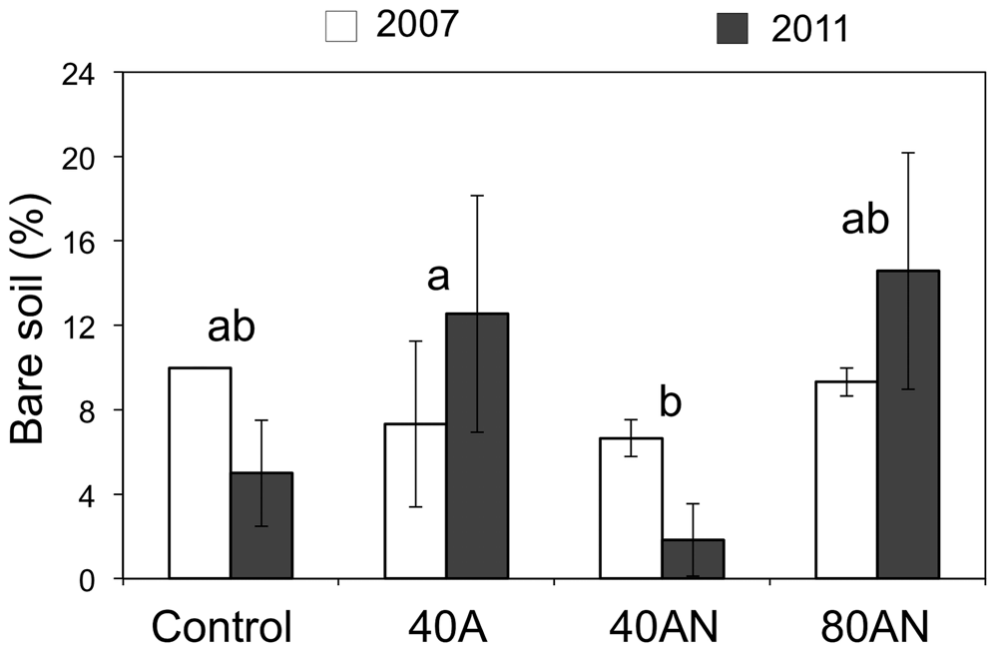
Impact of the N treatments on the % of bare soil. Response of the % of bare soil to the N treatments (Control, 40A, 40AN and 80AN) on the first and fifth springs of N additions (2007 and 2011). Different letters refer to statistically significant differences between treatments (two-way ANOVA *p*<0.1 followed by a Bonferroni test; there were no significant interactions between treatment and time). Bars represent the mean (n = 3 experimental plots per treatment) ±SE.

Initially control plots had most bare ground (Fig. 5), but by the fifth spring of the experiment, differences between treatments were visible: addition of 40AN reduced the area of bare ground whereas 40A increased it.

## Discussion

### N-driven enhanced plant richness: is the impact transient?

To our knowledge, this is the first integrated study of the impacts of different N doses and forms on a Mediterranean Basin ecosystem. In this post-fire successional ecosystem, addition of N continued to promote vascular plant richness, even after 5 years. Assuming that, in this ecosystem, N availability is a severe environmental limitation, the N-driven increase in richness may be explained by the revised Grime's humped-back model [37], [38]. The richness increment would reflect the initial alleviation of the stress condition (N limitation), allowing the coexistence of the characteristic site species [35], [39] with the incoming exploitative ones [40], [41]. The sustained increase in plant richness during the 5 years of N additions may be due to four non-exclusive causes:

- The difference in life span between the N-benefited (with short life cycles, including annuals) and the N-affected species (perennials), which would allow the detection of the incomers but only drastic impacts on the losers;
- The ecology of the N-benefited species would allow them to colonize the increased bare soil left by the death of some perennial N-affected plants (*e.g. C. ladanifer* [29]);
- The ecosystem is also limited by lack of water and phosphorus (P), so that the most aggressive/competitive species do not have the best conditions for their development. Not surprisingly, most N-benefited species were annuals that avoid summer drought as seeds but are highly dependent on adequate supplies of N and P [42], which can become more available with an improved N supply [20]. Even though it rained most in years 4 and 5 of the experiment, water alone cannot explain the enhanced plant richness as it did not increase in the control or 40AN plots;
- A large part of the added N is being lost from the ecosystem. This is an obvious hypothesis but one for which there is no supporting data (*i.e.* runoff, nitrate leaching, ammonia volatilization and denitrification were not measured). Although NO_3_^−^ leaching is considered to be an early indicator of N ‘saturation’ [8] in Mediterranean ecosystems, it has only been measured in Californian chaparral [43] and even then the N loss was less than expected due to immobilisation by the rapidly growing vegetation [44]. N accumulation in vegetation in that system was estimated to be 73 kg N ha^−1^ yr^−1^ [45], which is similar to our high N dose (80 kg N ha^−1^ yr^−1^). To quantify how much N the system could potentially ‘process’ before entering the descending phase of the humped model, longer-term studies are necessary.

The observed reduction in *C. ladanifer*'s cover [29] in this experiment probably resulted from its sensitivity to increased NH_4_^+^ availability [17], [26], even in the presence of high NO_3_^−^ availability [26]. The reduction of its canopy will have greatly increased light availability near the soil surface, allowing ‘exploitative’ species to establish themselves, increasing plant richness. In the presence of NO_3_^−^ but not too much NH_4_^+^ (40AN treatment), the overshading by *C. ladanifer* is likely to have excluded these small exploitative species. Keeping in mind that the range in NO_3_^−^ in our experiment is smaller than that of NH_4_^+^, the sensitivity of *C. ladanifer* to increased NH_4_^+^ availability would explain why plant richness was more related to the cumulative ammonium inputs than to the cumulative nitrate inputs.

This NH_4_^+^-driven increase in plant richness is unlikely to be observed when ecosystems are, or become, dominated by late successional species as these are more NH_4_^+^ tolerant [17], [26]. However, it is possible that climbing plants already present in the community (*e.g. Lonicera implexa*, *Rubia peregrina*, *Rubus ulmifolius*, etc.) may become dominant in later stages of succession, as in tropical ecosystems [6]. In conclusion, even though there are no data on the impacts of long-term enhanced N availability on Mediterranean Basin ecosystems, we consider that the N-driven increase in plant richness is transient.

### Are there consistent plant responders to increased N availability in Mediterranean maquis?

The composition of the plant community found at the experimental site was consistent with that expected in a Mediterranean maquis regenerating after a fire event [46]. After a fire, there is a ‘window of opportunity’, *i.e.*, a period of reduced competition for light, nutrients and water [40], [41], which favours the establishment of many annuals, hemicryptophytes, geophytes and subshrubs as well as ruderal species colonizing from the surroundings [46]. In a post-fire succession, as the ‘window of opportunity’ passes (∼5 years after the fire), some plant species tend to disappear from the community [47]. This natural dynamic of the plant community [22], [46] has to be taken into account as the background over which the population dynamics associated with the N additions have to be superimposed.

After one year, *D. viscosa* was the only species which had benefited from N addition (irrespective of dose and form). In subsequent years, its cover, together with that of *Sonchus* sp., began to decrease, which is consistent with their disappearance in late succession [40], [41], [48]. However, N additions appeared to offset some of the cover loss observed in the control, suggesting that increasing N availability extends the ‘window of opportunity’ for more nutrient-demanding plants. Other plant species responded more consistently to the N dose and/or form over the 5-year study period suggesting that they could be used as indicators of the N status of the ecosystem for at least a period of 5 years.

### Are the N-driven changes in diversity linked with functional alterations?

N-addition treatments failed to change soil C and N concentrations, or the C/N ratio, which remained within the range reported for the Mediterranean Basin [49]. In contrast to most studies [6], soil acidity was unaffected by the N additions, possibly due to the high soil calcium carbonate content [50]. However, after 5 years, application of the highest N dose (80AN) had increased soil extractable N, possibly indicating the transition from a closed N cycle to an open and leaky one where N may be lost through NO_3_^−^ leaching [51]. However, NO_3_^−^ leaching can occur much later than many diversity and internal N cycle changes [8]. On the other hand, and in contrast to observations of several studies [52], high N (80AN) addition does appear to have increased soil organic matter, most likely reflecting a decrease in decomposition [53]. This is of particular importance for Mediterranean Basin soils due to their naturally low organic matter concentration and hence higher susceptibility to erosion and desertification [54]. Altogether, it can be concluded that the cumulative N inputs have increased the ‘N status’ of the ecosystem [7], [55].

The aboveground biomass accumulated under control conditions was within the range of the equivalent Californian habitat – coastal sage scrub [36]. Under increased N availability, and in agreement with reports of other temperate [6] and Mediterranean ecosystems [18], [30], [56], aboveground plant biomass increased. Positive relationships between plant richness and productivity have been reported for Mediterranean [57] and several other ecosystems [58], [59]. However, in this study, plant richness and plant productivity were not related, as these were promoted by different N sources. The treatments with more NH_4_^+^ (40A and 80AN) promoted species richness, but the incomers were small plants that contributed very little to productivity or to protect the soil from erosion that may result from the increased % of bare soil. Given that the non-random N-driven loss of the dominant plant species has been shown to reduce productivity [3], the NH_4_^+^-driven reduction in the cover of the most abundant plant species (*e.g. Cistus ladanifer*, *Ulex densus*) would explain the absence of a productivity increase.

In general, N-enhanced plant richness was not linked with functional alterations, mainly because the N-benefited species were small short-lived plants, providing a minor contribution to ecosystem functions while the N-affected plants were perennial shrubs, which underpin ecosystem functions.

### Is the form of N more important than the dose?

The data suggest that plant community composition is driven by the amount of NH_4_^+^-N, highlighting the importance of NH_4_^+^ as a driving force in Mediterranean ecosystems [17], [26]. Given the characteristics of the Mediterranean climate (long dry periods interspersed with torrential rain events), which facilitates both rainfall and wind erosion [60], plant cover and the spatial structure of vegetation play a significant role in preventing soil loss and erosion in Mediterranean areas [61]. Due to the likely loss of cover with increasing NH_4_^+^ deposition, agricultural emissions are likely to increase erosion in the maquis. This suggests that enhanced NH_y_ availability (resulting mainly from agriculture) may affect the structure of the surrounding maquis, decrease soil protection (as a consequence of an increase in bare soil) and promote N leakage (as a consequence of an increase in soil extractable N).

In contrast, the lower NH_4_^+^-N dose applied to the combined N treatment (40AN) plots, with fewer plant species, improved aboveground biomass and soil protection most effectively by almost fully covering the soil with plants. Erosion can also be increased indirectly as a result of NO_3_^−^ deposition increasing aboveground biomass and the risk of wildfires, indicating that increasing urbanisation also threatens these ecosystems. Land managers and planners need to be aware of these effects before they authorise expansion of such N sources close to these ecosystems.

Overall, these results highlight the importance of cumulative N and enhanced N availability, particularly of NH_4_^+^, as a driving force behind the dynamics and stability in plant community structure and composition of Mediterranean ecosystems [17], [26]. The high resilience to disturbance of plant communities in the Mediterranean Basin has been related to their evolutionary history of disturbance, namely anthropogenic pressure [62]. Also, biodiversity may have its greatest impact on the functioning of diverse, naturally heterogeneous ecosystems [63], so it is possible that patchiness and heterogeneity may confer higher resilience to increased N availability (and hence higher reliability on ecosystem functions) than homogeneous un-fragmented ecosystems. Finally, the effectiveness of the critical N loads [64] established for this European habitat, at between 20 and 30 kg N ha^−1^ yr^−1^ [65] would be improved by inclusion of the impact of the N form.

## Supporting Information

Table S1 **Effect of the N treatments on plant community composition and cover.** List of the vascular plant species observed in the three assessments, and their respective changes in cover (2008–2007/2011–2007) according to the N additions.
(DOCX)

Table S2 **List of the plant species that responded consistently (after one and 5 years of N addition treatments) to the N dose and/or form.** The species' most common habitats and, when available, their responses to N enrichment in other studies are shown in the right column.
(DOCX)

Table S3 **Statistical analyses of soil surface properties.**
(DOCX)
