# Supplementary material for: Ammonium as a Driving Force of Plant Diversity and Ecosystem Functioning: Observations Based on 5 Years' Manipulation of N Dose and Form in a Mediterranean Ecosystem
Source: PLoS One. 2014 Apr 2;9(4):e92517. doi: 10.1371/journal.pone.0092517 (PMC3973647; doi:10.1371/journal.pone.0092517)
Supplement: Table S1 — Effect of the N treatments on plant community composition and cover. List of the vascular plant species observed in the three assessments, and their respective changes in cover (2008–2007/2011–2007) according to the N additions. (DOCX) [file pone.0092517.s001.docx]

**Table S1 – Effect of the N treatments on plant community composition and cover.**

| Life form | Family | | Species | Control | 40A | 40AN | 80AN |
| --- | --- | --- | --- | --- | --- | --- | --- |
| P (P) | Anacardiaceae | | *Pistacia lentiscus* | -/0 | -/0 | .+/+ | -/0 |
|  | Caprifoliaceae | | *Lonicera implexa* |  | /(+) |  |  |
|  | Cistaceae | | *Cistus crispus* | /(+) | /(+) | (-)/+ | +/- |
|  |  | | *Cistus ladanifer* | .+ a/+ a | .+ a/+ ab | .+ a/+ a | .- b/- b |
|  |  | | *Cistus monspeliensis* |  | +/- |  | (+)/(+) |
|  |  | | *Cistus salvifolius* | +/+ | +/- | +/0 | -/- |
|  |  | | *Halimium halimifolium* | +/0 |  |  |  |
|  | Ericaceae | | *Arbutus unedo* |  | /(+) |  | -/0 |
|  |  | | *Calluna vulgaris* | +/+ | +/0 | +/+ | +/- |
|  |  | | *Erica arborea* |  | +/+ | +/+ |  |
|  |  | | *Erica scoparia* | +/+ | +/+ | +/+ | +/+ |
|  |  | | *Erica umbellata* |  |  |  | .+/+ |
|  | Fabaceae | | *Genista triacanthos* | +/+ | +/+ | +/+ | 0/- |
|  |  | | *Ulex densus* | +/0 a | -/- b | +/+ a | +/+ a |
|  | Fagaceae | | *Quercus coccifera* |  | .+/(-) | +/+ | 0/+ |
|  |  | | *Quercus lusitanica* | /(+) |  |  | /(+) |
|  |  | | *Quercus suber* |  |  | 0/0 |  |
|  | Lamiaceae | | *Lavandula stoechas* | +/0 | -/- | +/+ | 0/+ |
|  |  | | *Rosmarinus officinalis* |  | (+)/(+) | /(+) | +/0 |
|  | Myrtaceae | | *Myrtus communis* | +/0 | 0/0 | +/0 | -/0 |
|  | Oleaceae | | *Olea europaea* var *sylvestris* |  |  | .+/0 | +/0 |
|  |  | | *Phillyrea angustifolia* | /(+) |  | /(+) | .+/+ |
|  | Pinaceae | | *Pinus pinaster* |  | -/0 |  | +/0 |
|  | Rhamnaceae | | *Rhamnus alaternus* |  |  | .+/+ | /(+) |
|  | Rosaceae | | *Prunus* sp |  | /(+) |  | /(+) |
|  |  | | *Rubus ulmifolius* | (-)/0 | (+)/(+) | -/+ |  |
|  | Thymelaeaceae | | *Daphne gnidium* | 0/0 | .+/+ | -/- | (+)/(+) |
| P (C) | Asteraceae | | *Dittrichia viscosa* | .- b/- | .+ a/0 | .+ a/- | .+ a/- |
|  |  | | *Helichrysum stoechas* | (-)/(-) | /(+) |  | -/- |
|  |  | | *Phagnalon saxatile* |  | /(+) |  | (-)/- |
|  | Boraginaceae | | *Lithodora prostrata* | 0/0 |  | +/0 | (+)/ |
|  | Lamiaceae | | *Thymus villosus* |  |  |  | /(+) |
|  | Rubiaceae | | *Rubia peregrina* |  | /(+) |  | (+)/(+) |
| P (G) | Apiaceae | | *Conopodium* sp |  |  | /(+) |  |
|  | Asphodelaceae | | *Asphodelus ramosus* | (-)/(-) |  |  | (+)/(+) |
|  | Asteraceae | | *Carlina corymbosa* |  | /(+) | (+)/(+) | /(+) |
|  | Hyacinthaceae | | *Urginea maritima* | /(+) | (+)/(+) |  | (-)/- |
|  | Iridaceae | | *Gladiolus illyricus* ssp *reuteri* |  | (+)/(+) | (+)/ | (+)/(+) |
|  |  | | *Iris xiphium* |  |  |  | (+)/ |
|  | Orobanchaceae | | *Orobanche latisquama* | (+)/ |  |  |  |
|  | Ranunculaceae | | *Anemone palmata* | (+)/(+) |  | /(+) |  |
| P (H) | Apiaceae | | *Daucus carota* |  |  | (-)/(-) |  |
|  | Asteraceae | | *Andryala ragusina* | -/0 |  |  |  |
|  |  | | *Cynara* sp | (-)/- | /(+) | /(+) |  |
|  |  | | *Leontodon taraxacoides* | -/- | -/- | +/- | 0/- |
|  |  | | *Pulicaria odora* |  |  | (-)/0 | (+)/(+) |
|  |  | | *Senecio jacobaea* | (+)/ |  | (-)/(-) |  |
|  | Campanulaceae | | *Campanula rapunculus* |  | 0/+ | (-)/(-) | /(+) |
|  | Cyperaceae | | *Carex flacca* | +/- | +/+ | +/+ | -/- |
|  | Fabaceae | | *Anthyllis vulneraria* |  |  |  | (+)/ |
|  | Gentianaceae | | *Blackstonia perfoliata* | -/- | -/- | (-)/- | 0/+ |
|  |  | | *Centaurium erythraea* | -/- | -/- | (-)/(-) | -/- |
|  | Hypericaceae | | *Hypericum* sp | -/- b | -/- ab | -/- a | /(+) a |
|  | Lamiaceae | | *Prunella laciniata* | /(+) |  |  |  |
|  |  | | *Salvia sclareoides* | (-)/(-) |  |  | (+)/(+) |
|  | Poaceae | | *Agrostis* sp | (+)/ |  |  |  |
|  |  | | *Arrhenatherum album* |  | +/(-) |  | (-)/(-) |
|  |  | | *Avenula* sp | (+)/ | /(+) |  | /(+) |
|  |  | | *Brachypodium phoenicoides* | -/(-) | 0/0 | -/- | +/+ |
|  |  | | *Dactylis glomerata* |  |  |  | (+)/(+) |
|  | Rosaceae | | *Sanguisorba hybrida* | /(+) | /(+) | (-)/- | (-)/(-) |
| nd(H/T) | Asteraceae | | *Crepis capillaris* |  | (+)/ |  |  |
|  |  | | *Picris echioides* | /(+) | (-)/+ |  | +/+ |
|  | Fabaceae | | *Lotus sp* | (+)/(+) | (+)/(+) | +/- | /(+) |
|  |  | | *Trifolium* sp | (-)/- |  | (-)/(-) | (-)/(-) |
|  |  | | *Vicia* sp |  | (+)/ |  |  |
|  | Lythraceae | | *Lythrum* sp |  | /(+) |  |  |
| A (T) | Asteraceae | | *Centaurea melitensis* | (+)/ |  |  | /(+) |
|  |  | | *Crysanthemum coronarium* |  | (+)/ |  |  |
|  |  | | *Evax pygmaea* |  |  |  | (+)/ |
|  |  | | *Filago minima* | -/- | (-)/0 | (+)/ | (-)/- |
|  |  | | *Galactites tomentosa* | -/(-) | (-)/- | (-)/(-) | (-)/- |
|  |  | | *Matricaria recutita* | (-)/(-) | (-)/(-) | (-)/(-) |  |
|  |  | | *Sonchus* sp | (-)/(-) b | /(+) a | (-)/0 b | (+)/(+) b |
|  | Campanulaceae | | *Solenopsis laurentia* | /0 | /(+) |  | /(+) |
|  | Lamiaceae | | *Stachys arvensis* | (+)/ | (+)/ | (+)/ |  |
|  | Poaceae | | *Briza maxima* |  |  | (+)/(-) | (+)/ |
|  |  | | *Briza minor* | (-)/(-) | (-)/ | 0/(-) | (+)/ |
|  |  | | *Gastridium ventricosum* | /(+) b | /(+) a |  | /(+) b |
|  | Primulaceae | | *Anagallis arvensis* | (+)/(+) | -/- | +/- | +/0 |
|  | Scrophulariaceae | | *Kickxia cf. cirrhosa* | /(+) |  | /(+) | /(+) |
| nd | Apiaceae | | nd | (+)/ |  | /(+) | (+)/(+) |
|  | Asteraceae | | nd | (-)/(-) | (-)/(-) | (+)/ | /(+) |
|  | Boraginaceae | | nd |  |  |  | /(+) |
|  |  | | *Echium* sp |  | /(+) |  |  |
|  | Cruciferae | | nd |  | /(+) |  |  |
|  | Fabaceae | | nd |  |  |  | (-)/(-) |
|  | Liliaceae | | nd | (+)/ |  | (+)/ |  |
|  | Malvaceae | | nd | /(+) |  |  |  |
|  | Poaceae | | nd | 0/- | /(+) | .-/(-) | 0/- |
|  |  | | *Vulpia* sp | (-)/(-) | (-)/(-) |  | (-)/(-) |
|  | Rubiaceae | | *Galium* sp |  | /(+) | (+)/ | (+)/(+) |
|  |  | | nd |  |  |  | (+)/ |
| Number of | | sp that: | Increased + | .10/5 | .12/8 | .19/13 | .13/9 |
|  |  | | Decreased - | .10/10 | .9/10 | .5/9 | .8/15 |
|  |  | | Maintained 0 | .3/10 | .3/7 | .2/7 | .7/7 |
|  |  | | Appeared (+) | .11/13 | .9/24 | .8/8 | .17/26 |
|  |  | | Disappeared (-) | .11/10 | .7/5 | .12/10 | .9/5 |

List of the vascular plant species observed in the three assessments (the first, second and fifth springs of N additions in June 2007, May 2008 - [1] - and June 2011), and their respective changes in cover (2008-2007 / 2011-2007) according to N additions (Control, 40A, 40AN and 80AN). Changes in plant species cover over time between 2007 and 2011 were calculated. Changes between 2007 (the first spring of N additions – t_1_) and 2008 or 2011 (the second and fifth spring of N additions – t_2_, t_5_) were positive (an increase), negative (a decrease) or null (no change) and were calculated as follows [1,2]:

Plant species are grouped according to their life form: the first letter in each line of the column “life form” represents perennial (P), annual (A) or undetermined (nd); and the second letter represents the biological type: phanerophyte (P), chamaephyte (C), geophyte (G), hemicryptophyte (H), therophyte (T) or undetermined (nd). The subdivisions of the Raunkier system [3] are based on the location of the plant's growth-point (bud) during seasons with adverse conditions: phanerophytes (P) – usually woody perennials that project their buds on stems more than 25 cm above soil level; chamaephytes (C) – woody plants with perennating buds appearing close to the ground; hemicryptophytes (H) – with buds at or near the soil surface; geophytes (G) – with buds resting in the soil; and therophytes (T) – annual plants which survive the unfavourable season in the form of seeds. The columns describing the effects of the treatments for the two periods 2008-2007 and 2011-2011 indicate: +, increased cover; 0, no change in cover; -, decreased cover; (+), species appearance; and (-), species disappearance. Different letters refer to significant differences between treatments (ANOVA *p*<0.1 followed by a Bonferroni test and Kruskal-Wallis *p*<0.1 for normal and non-normal distributions respectively; n = 3 experimental plots per treatment).

References:

1. Dias T, Malveiro S, Martins-Loucao MA, Sheppard LJ, Cruz C (2011) Linking N-driven biodiversity changes with soil N availability in a Mediterranean ecosystem. Plant and Soil 341: 125-136.

2. Sheppard LJ, Leith ID, Crossley A, Van Dijk N, Fowler D, et al. (2008) Stress responses of *Calluna vulgaris* to reduced and oxidised N applied under 'real world conditions'. Environmental Pollution 154: 404-413.

3. Raunkier C (1934) The life forms of plants and statistical plant geography, being the collected papers of C. Raunkier. Oxford: Oxford University Press.
