# Supplementary material for: Ammonium as a Driving Force of Plant Diversity and Ecosystem Functioning: Observations Based on 5 Years' Manipulation of N Dose and Form in a Mediterranean Ecosystem
Source: PLoS One. 2014 Apr 2;9(4):e92517. doi: 10.1371/journal.pone.0092517 (PMC3973647; doi:10.1371/journal.pone.0092517)
Supplement: Table S2 — List of the plant species that responded consistently (after one and 5 years of N addition treatments) to the N dose and/or form. The species' most common habitats and, when available, their responses to N enrichment in other studies are shown in the right column. (DOCX) [file pone.0092517.s002.docx]

**Table S2 – List of the plant species potentially indicative of the N dose and form.**

| N treatments | | | | Plant species | Ecology/habitat |
| --- | --- | --- | --- | --- | --- |
| N dose | Low N |  |  | None |  |
|  | High N | Short-term |  | *Dittrichia viscosa* | Roadsides, abandoned fields and walls [1] |
|  |  |  |  | *Sonchus* sp | Agricultural and abandoned fields (Flora Digital de Portugal); an exotic invader in Californian high N areas [2] |
|  |  | Longer-term | Medium and high N | *Carlina corymbosa* | Abandoned and disturbed fields (Flora Digital de Portugal) |
|  |  |  |  | *Gladiolus illyricus* ssp *reuteri* | Shrublands and abandoned fields (Flora Digital de Portugal) |
|  |  |  |  | *Galium* sp | Agricultural and abandoned/degraded fields, sometimes rupicolous (Flora Digital de Portugal) |
|  |  |  | Very high N | *Salvia sclareoides* | Abandoned and disturbed areas (Flora Digital de Portugal) such as degraded communities of *Juniperus* [3] |
|  |  |  |  | *Asphodelus ramosus* | Shrublands and abandoned areas (Flora Digital de Portugal); dominant in many degraded Mediterranean areas [4] |
|  |  |  |  | *Blackstonia perfoliata* | Grasslands (Flora Digital de Portugal); benefits from cutting management [5] |
|  |  |  |  | *Dactylis glomerata* | Grasslands, abandoned fields and disturbed areas (Flora Digital de Portugal) |
|  |  |  |  |  |  |
| N form | NO_3_^-^ | Benefited |  | *Pulicaria odora* | Abandoned areas and shrublands (Flora Digital de Portugal) |
|  |  | Affected |  | *Sanguisorba hybrida* | Abandoned areas and shrublands (Flora Digital de Portugal) and *S. minor*; is associated with high N deposition [6] |
|  | NH_4_^+^ | Benefited |  | *Rubia peregrina* | Shrublands and rupicolous (Flora Digital de Portugal) |
|  |  |  |  | *Brachypodium*  *phoenicoides* | Shrublands and abandoned areas (Flora Digital de Portugal) |
|  |  | Affected |  | *Anemone palmata* | Grasslands, shrublands and rupicolous (Flora Digital de Portugal) |

List of the plant species that responded consistently (after one and 5 years of N addition treatments) to the N dose and/or form (Table S1). The species’ most common habitats and, when available, their responses to N enrichment in other studies are shown in the right column. “Low N” refers to ambient N deposition, *i.e.*, <4 kg N ha^-1^ yr^-1^; “High N” refers to the N treatments. Considering the longer-term, *i.e.*, 5 years, “Medium and high N” refers to the 40 kg N ha^-1^ yr^-1^ treatments, while “Very high N” refers to the 80 kg NH_4_NO_3_-N ha^-1^ yr^-1^ treatment.

References:

1. Brullo S, Marco G (2000) Taxonomical revision of the genus *Dittrichia* (Asteraceae). Portugaliae Acta Biologica 19: 341-354.

2. Cione NK, Padgett PE, Allen EB (2002) Restoration of a native shrubland impacted by exotic grasses, frequent fire, and nitrogen deposition in southern California. Restoration Ecology 10: 376-384.

3. Costa JC, Lousã M, Capelo J, Santo MDE, Sevillano JI, et al. (2000) The coastal vegetation of the Portuguese divisory sector: dune cliffs and low-scrub communities. Finisterra XXXV 69: 69-93.

4. Sakar FS, Arslan H, Kirmizi S, Guleryuz G (2010) Nitrate reductase activity (NRA) in Asphodelus aestivus Brot. (Liliaceae): distribution among organs, seasonal variation and differences among populations. Flora 205: 527-531.

5. Bonanomi G, Caporaso S, Allegrezza M (2006) Short-term effects of nitrogen enrichment, litter removal and cutting on a Mediterranean grassland. Acta Oecologica-International Journal of Ecology 30: 419-425.

6. Stevens CJ, Smart SM, Henrys P, Maskell LC, Walker KJ, et al. (2011) Collation of evidence of nitrogen impacts on vegetation in relation to UK biodiversity objectives. Joint Nature Conservation Committee.
