# Supplementary material for: Ammonium as a Driving Force of Plant Diversity and Ecosystem Functioning: Observations Based on 5 Years' Manipulation of N Dose and Form in a Mediterranean Ecosystem
Source: PLoS One. 2014 Apr 2;9(4):e92517. doi: 10.1371/journal.pone.0092517 (PMC3973647; doi:10.1371/journal.pone.0092517)
Supplement: Table S3 — Statistical analyses of soil surface properties. (DOCX) [file pone.0092517.s003.docx]

**Table S3 – Statistical analyses of soil surface properties.**

| Variable | Time | | | Treatment | | | Time x Treatment | | |
| --- | --- | --- | --- | --- | --- | --- | --- | --- | --- |
|  | df | F | P | df | F | P | df | F | P |
| N | 1 | 0.5 | 0.491 | 3 | 1.2 | 0.332 | 3 | 0.0 | 0.989 |
| C | 1 | 1.1 | 0.305 | 3 | 0.6 | 0.597 | 3 | 0.0 | 0.989 |
| C/N ratio | 1 | 1.2 | 0.292 | 3 | 1.2 | 0.340 | 3 | 0.1 | 0.965 |
| N_in_ | 1 | 10.5 | **0.005** | 3 | 6.3 | **0.005** | 3 | 1.4 | 0.274 |
| NO_3_^-^-N | 1 | 10.8 | **0.005** | 3 | 4.9 | **0.013** | 3 | 2.4 | 0.109 |
| NH_4_^+^-N | 1 | 1.1 | 0.311 | 3 | 6.4 | **0.005** | 3 | 1.4 | 0.269 |
| OM | 1 | 0.6 | 0.440 | 3 | 4.2 | **0.023** | 3 | 0.8 | 0.515 |
| pH | 1 | 35.7 | **0.000** | 3 | 2.0 | 0.153 | 3 | 1.2 | 0.356 |

Soil surface (0-15 cm) properties [total N and C, C/N ratio, concentrations of N_in_ – extractable inorganic N, NO_3_^-^ – nitrate, NH_4_^+^ – ammonium, OM – organic matter, and pH (H_2_O) determined in a water extract] at the time of sampling and treatments (Control, 40A, 40AN and 80AN). A two-way ANOVA was conducted (*p*<0.05 are shown in bold).
